# A report of 2 cases of Cornelia de Lange syndrome (CdLS) and an analysis of clinical and genetic characteristics in a Chinese CdLS cohort

**DOI:** 10.1002/mgg3.1066

**Published:** 2019-12-24

**Authors:** Shuo Li, Hui Miao, Hongbo Yang, Linjie Wang, Fengying Gong, Shi Chen, Huijuan Zhu, Hui Pan

**Affiliations:** ^1^ Key Laboratory of Endocrinology of National Health and Family Planning Commission Department of Endocrinology Peking Union Medical College Hospital Chinese Academy of Medical Science and Peking Union Medical College Beijing China

**Keywords:** Chinese CdLS patients, Cornelia de Lange syndrome, *NIPBL* mutation, scoring system

## Abstract

**Background:**

Cornelia de Lange syndrome (CdLS) is a rare dominantly inherited developmental disorder with an estimated prevalence of 0.5–10:100,000 and no racial disparity in prevalence. The aim of this study was to present two unrelated Chinese CdLS individuals with mutations in *NIPBL* and to perform a comprehensive analysis of a Chinese cohort with CdLS.

**Subjects and methods:**

Two unrelated Chinese patients complaining of short stature were referred to the outpatient department of Peking Union Medical College Hospital (PUMCH). Their clinical data at birth and at the most recent assessment were collected. Mutation analysis was carried out by whole exome sequencing. Twenty‐four Chinese cases with CdLS were identified through a systematic review of the literature published between 1987 and 2017.

**Results:**

Two patients presented with typical phenotypes, characteristic complications of CdLS and mutations in the *NIPBL* gene. The average age at diagnosis of the 26 Chinese cases was higher than that of other cohorts. The frequencies of characteristic manifestations of CdLS were similar with those of other populations.

**Conclusions:**

By investigating 26 Chinese cases of CdLS, we observed that the clinical data and gene variants in the Chinese cohort of CdLS patients were generally in accordance with those of other populations.

## Graphical abstract

We found two patients presented with typical phenotypes, characteristic complications of CdLS and mutations in the NIPBL gene. By investigating 26 Chinese cases of CdLS, we observed that the clinical data and gene variants in the Chinese cohort of CdLS patients were generally in accordance with those of other populations. To date, the NIPBL‐LOVD database (Leiden open variation database) describes 266 unique DNA variants, reported in 352 individuals (last accessed July 2017) which include substitutions (65.1%, 229/352), deletions (23.9%, 84/352), duplications (8.8%, 31/352), insertions/deletions (1.4%, 5/352) and insertions (0.9%, 3/352) (Figure 4).

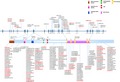

## 1 INTRODUCTION

Cornelia de Lange syndrome (CdLS, also called Brachmann‐de Lange syndrome, Online Mendelian Inheritance in Man (OMIM) entries 122,470, 300,590, 300,882, 610,759 and 614,701), which was initially reported Brachmann (1916) and further characterized by de Lange (1933), is a rare genetically heterogeneous disorder affecting a wide range of tissues and organs. The characteristic manifestations of CdLS include facial dysmorphia, growth retardation, psychomotor delay, abnormality of the upper limbs, major malformations (especially microcephaly and malformations of the cardiac, genital, gastrointestinal and urological systems) and medical complications (ocular defects, gastrointestinal reflux, neurosensory deafness, etc.). Heterozygous mutations in five genes (*NIPBL, SMC1A, SMC3, HDAC8,* and *RAD21*) have been found to be contributory to CdLS, with *NIPBL* gene (NM_133433.3, NG_006987.2 RefSeqGene) mutations responsible for approximately 50% of CdLS cases (Mannini, Cucco, Quarantotti, Krantz, & Musio, 2013).

Herein, we present two unrelated Chinese CdLS cases. Furthermore, the clinical characteristics of Chinese CdLS cases published previously were summarized and compared with those of other populations.

## 2 PATIENTS AND METHODS

### 2.1 Patients and clinical evaluation

#### 2.1.1 Ethical compliance

Examination protocols were approved by the ethics committees of Chinese Academy of Medical Science and Peking Union Medical College with the following reference number: JS‐1663. Written informed consents were obtained from the subjects for the publication of the case report and any accompanying images.

Two patients with CdLS‐like phenotypes from two unrelated Chinese families were referred to the outpatient department of Peking Union Medical College Hospital (PUMCH). Demographic parameters were requested at birth and at the most recent assessment. No parental consanguinity or positive family history of any deformity was found in any of the cases. All subjects underwent complete evaluation, focusing on psychomotor development, malformations or medical complications.

### 2.2 Mutation analysis by whole exome sequencing

#### 2.2.1 Exome sequencing protocol

Two‐milliliter peripheral blood samples were taken from two patients. Genomic DNA was isolated using the QIAamp DNA Blood Mini Kit. Two micrograms of DNA were fragmented randomly to an average size of 250 bp using a Covaris acoustic system. An adapter‐ligated library was pooled and hybridized with a BGI in‐house 59M whole exome capture kit to capture the target regions. Quantitative PCR was performed to estimate the magnitude of enrichment. Then, the qualified captured library was sequenced on a HiSeq 2,500 analyzer (Illumina, Inc.) for a 100‐bp paired‐end run.

#### 2.2.2 Read alignment, variant calling, and annotation

Illumina Pipeline (version 1.3.4) was used for image analysis, error estimation, base calling and generating the primary sequence data. The clean data containing paired‐end reads were mapped to the human genome (NCBI37/hg19) using BWA software (Burrows Wheeler Aligner, http://sourceforge.net/projects/bio-bwa/). SNVs (single nucleotide variants) and small InDels (insertion and deletions) were identified by SOAP Snp software (http://soap.genomics.org.cn/) and SAMtools Pileup software (http://sourceforge.net/projects/samtools/), respectively. The variants were annotated using Gaea, a BGI in‐house‐developed annotation pipeline. The public databases used to calculate the frequency in the normal population included the 1K genome database (http://www.1000genomes.org/), ESP6500 database, dbSNP database and BGI in‐house database. The software used for nonsynonymous functional predictions included PolyPhen‐2, SIFT and Ens Condel.

### 2.3 Literature review

We systematically searched the online literature databases Pubmed, Embase, Medline, Wanfang, China National Knowledge Infrastructure (CNKI) and the Cochrane Collaboration Library for articles associated with a Chinese cohort of CdLS patients. Finally, 24 previously reported cases of CdLS in the Chinese population were identified (Table 1). All subjects in the included papers complied with the genetic sequencing diagnosis or clinical diagnostic criteria for CdLS suggested by the CdLS Foundation's Medical Director, Kline et al. (2018).

**Table 1:** Clinical parameters and genetic analysis of 26 cases with CdLS so far reported in China. Y, year; m, month; SDS, standard deviation score; NB, newborn; UM, unmeasured

Patient	Gender/age	Height at test (cm)	Birth weight (kg)	Intrauterine growth retardation	Post‐natal retardation	Facial dysmorphisms	Skeletal deformations	Mental retardation	Neurosensory manifestations	Gastrointestinal manifestations	Cardiovascular manifestations	Genitourinary manifestations	Skin manifestations	Clinical score	Sequence change (AA change)
1	M/NB	43 (<−3SD)	2.5 (−2SD–3SD)	+	+	+	+	+	−	−	−	+	+	16‐Classic	UM
2	F/8m	53 (<−3SD)	1.7 (<−3SD)	+	+	+	+	+	+	+	−	−	+	12‐Classic	UM
3	F/5m	58 (<−3SD)	2.3 (−2SD–3SD)	+	+	+	+	+	−	−	−	−	+	12‐Classic	UM
4	M/3y	87 (−2<−3SD)	2.15 (<−3SD)	+	+	+	+	+	−	−	−	+	–	12‐Classic	UM
5	F/6m	55 (<−3SD)	2.05 (<−3SD）	+	+	+	+	+	−	+	+	−	+	7‐sufficient for molecular testing	UM
6	F/3y	80 (<−3SD)	2.3 (−2SD–3SD)	+	+	+	+	+	−	−	−	−	+	15‐classic	UM
7	M/17m	58 (<−3SD)	2 (<−3SD)	+	+	+	+	+	−	−	−	+	+	15‐classic	UM
8	F/NB	40 (<−3SD)	1.65 (<−3SD)	+	+	+	+	+	+	+	−	−	+	7‐sufficient for molecular testing	UM
9	F/1m	48 (<−3SD)	1.95 (<−3SD)	+	+	+	+	+	−	−	−	−	+	12‐classic	UM
10	M/NB	42 (<−3SD)	1.8 (<−3SD)	+	+	+	+	+	−	−	−	−	+	12‐classic	UM
11	F/NB	42 (<−3SD)	1.73 (<−3SD)	+	+	+	+	+	−	−	−	−	+	10‐classic	UM
12	M/NB	40 (<−3SD)	1.25 (<−3SD)	+	+	+	+	+	−	−	+	+	+ß	10‐classic	UM
13	M/6y	98 (<−3SD)	2.6 (−SD–2SD)	−	+	+	+	+	+	+	+	+	+	12‐classic	UM
14	F/NB	42 (<−3SD)	2.04 (<−3SD)	+	+	+	+	+	−	−	+	+	+	6‐sufficient for molecular testing	UM
15	F/1y	61 (<−3SD)	2.2 (<−3SD)	+	+	+	+	+	−	+	−	−	+	13‐classic	UM
16	M/2.5y	80.4 (<−3SD)	2.19 (<−3SD)	+	+	+	+	+	−	+	−	+	−	14‐classic	UM
17	M/13 y	133.5 (<−3SD)	2.16 (<−3SD)	+	+	+	+	+	−	+	−	−	−	12‐classic	UM
18	F/NB	38 (<–3SD)	1.63 (<−3SD)	+	+	+	+	+	+	−	+	−	+	12‐classic	UM
19	M/2.8 y	67 (<−3SD)	1.9 (<−3SD)	+	+	+	+	+	−	−	+	+	−	11‐classic	c.7176T > A (p.Cys2392Ter) Exon 42
20	F/8 m	58 (<−3SD)	1.6 (<−3SD)	+	+	+	+	+	+	−	−	−	−	10‐non‐classic	UM
21	M/5.5 y	96 (<−3SD)	2.6 (−SD–2SD)	−	+	+	+	+	+	+	−	−	−	13‐classic	normal
22	F/2.8 y	76 (<−3SD)	2.75 (−SD–2SD)	−	+	+	+	+	−	+	−	−	−	12‐classic	c.4321G > T(p.Val 1441Leu) Exon 20
23	F/2 y	74 (<−3SD)	2.5 (−2SD–3SD)	+	+	+	+	−	−	+	+	−	+	14‐classic	c.6589 + 5G>C Intron 38
24	F/2 m	60 (<−3SD)	3	−	−	+	+	+	+	−	+	−	+	11‐classic	normal
25	F/17 y	134.3 (−2SD<−3SD)	2.5 (−2SD–3SD)	+	+	+	+	+	−	−	−	−	−	9‐non classic	c.8274_8275ins CT (p. Val2760 Trpfs) Exon 47

Note

Based on the scoring system of clinical phenotype of CdLS proposed by Kline et al. (2018). classic CdlS: ≥11 points, of which at least 3 are cardinal; non‐classic CdlS: 9 or 10 points, of which at least 2 are cardinal; sufficient to indicate molecular testing for CdlS: 4–8 points, of which at least 1 is cardinal; insufficient to indicate molecular testing for CdlS: <4 points.

### 2.4 Applying the consensus criteria of clinical phenotype in Chinese patients with CdLS

Each individual was evaluated by the consensus criteria of clinical phenotype of CdLS, which integrates facial dysmorphisms, growth retardation, psychomotor development, hand oligodactyly and/or adactyly, hirsutism and other major malformations (Kline et al., 2018). The patients are classified into four phenotypes. Individuals having at least three cardinal features and an overall score of ≥11 are considered as “classic CdLS” phenotype. An overall score of 9–10 with at least two cardinal features indicates “non‐classic CdLS”. A score of ≥4 and at least one cardinal feature is considered as being sufficient to indicate molecular testing for CdLS. A score of <4 is insufficient to conduct such testing. No other cohorts of Chinese CdLS patients have been investigated with this consensus criteria of clinical phenotypes.

## 3 RESULTS

### 3.1 Analysis of clinical characteristics

#### 3.1.1 Clinical data of the two patients in the present study

Both of the two patients in the present study were children of clinically asymptomatic nonconsanguineous parents with normal height and no genetic defects.

**Figure 1:**
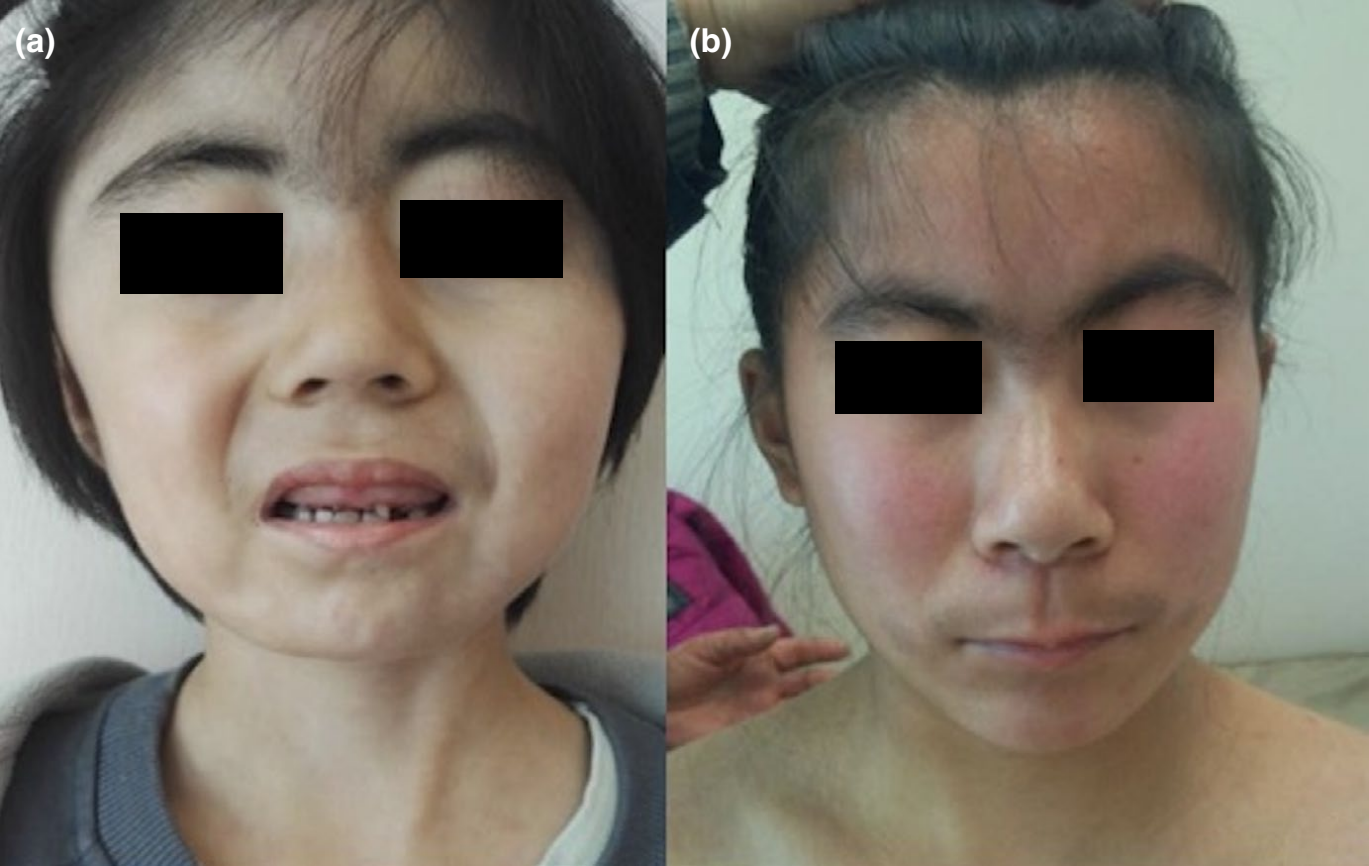
Facial abnormalities of patient 1(a) and 2(b) referring to the outpatient department of PUMCH [Correction added on 02 March 2020, after first online publication: in Figure1, the patients' eyes have been hidden.]

Patient 1 (Figure 1a) was born at a gestational age of 34 weeks by cesarean section. At birth, her weight was 2.15 kg. At the age of 5 years, her height was 100 cm (−2.43 SDS). Her bone age was unmeasured. She manifested with a low hair line, synophrys, hypertrichosis of the brow and long curly eyelashes. She had bone anomalies that included 5th clinodactyly in the fingers of both hands and 2nd and 3rd syndactyly in the toes of both feet. She also presented microcephaly, patent ductus arteriosus and dental anomalies. Her serum IGF‐1 level was 73.8 ng/ml (−1.72 SDS) and GH level was 16.3 ng/ml.

**Figure 2:**
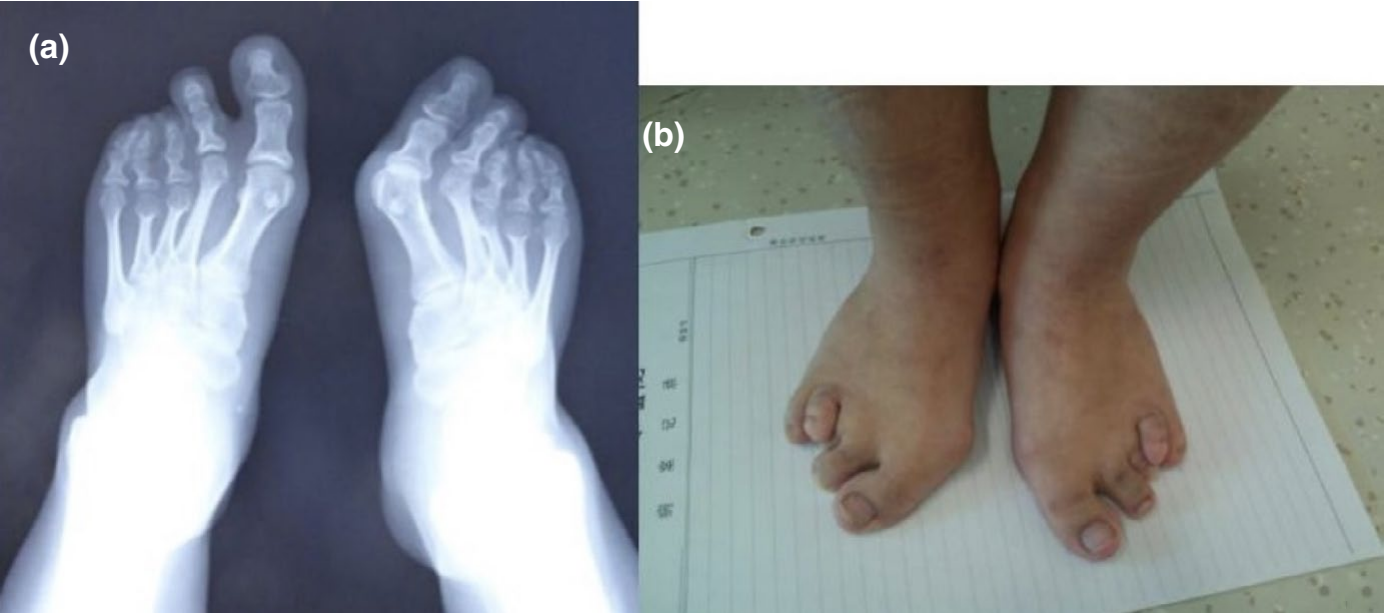
Bone anomalies of patient 2 (a: skeletal X ray image of both feet)

Patient 2 (Figure 1b) was born full‐term by a normal delivery, with a birth weight of 2.5 kg. She was 17 years old, and her height was 134.3 cm (−4.81 SDS). Her bone age was 16–18 years. She had synophrys and long eyelashes. She also exhibited bone anomalies, including 5th clinodactyly in the fingers of both hands and 3rd and 4th syndactyly in the toes of both feet (Figure 2). In addition, she had impaired expressive communication and learning disabilities. Her serum IGF‐1 was 351 ng/ml (−1.52 SDS). No abnormalities were found in ophthalmologic examination, echocardiography or other evaluations.

#### 3.1.2 Clinical characteristics of 26 reported Chinese CdLS cases

We collected data on a group of 24 Chinese cases of CdLS from the literature and analyzed characteristics of the total 26 Chinese cases including 2 cases in the current study. The 26 cases were classified into the “classic” (69.2%, 18/26), “non‐classic” (15.4%, 4/26) and “being sufficient to indicate molecular testing for CdLS” (15.4%, 4/26) phenotype subgroups according to the scoring system.

The mean age at clinical diagnosis was 2.85 years (range: birth to 17 years). The clinical diagnosis was established within the neonatal period in 30.8% of cases (8/26). 23.1% (6/26) of the cases were diagnosed later, but within the first year of life. Gestational duration was unknown in 19.2% (5/26) of the cases, while 28.6% (6/21) of the cases were preterm and 71.4% (15/21) were full‐term at birth. Prenatal growth retardation was evident in 88.5% (23/26) of the patients. The mean birth weight was 2,130 g. Among these cases, 73.1% (19/26) were low‐birth‐weight infants (LBWIs) (1.5–2.5 kg), and 3.85% (1/26) were very‐low‐birth‐weight infants (VBIWIs) (1–1.5 kg).

All of the patients had the typical craniofacial characteristics of CdLS, including synophrys (92.3%, 24/26); hypertrichosis of the brows (92.3%, 24/26); long eyelashes (84.6%, 22/26); hirsutism (69.2%, 18/26); thin lips with down‐turned corners (69.2%, 18/26); a broad and depressed nasal bridge (65.4%, 17/26); a low hair line (61.5%, 16/26); a high arched palate (57.7%, 15/26); low‐set ears (42.3%, 11/26); a long, shallow and prominent philtrum (42.3%, 11/26); and micrognathia (38.5%, 10/26).

96.2% of the cases (25/26) had limb involvement, which included single palmar creases (65.4%, 17/26), small hands with short and thin finger tips (53.8%, 14/26), 5th finger clinodactyly (38.5%, 10/26), hypophalangism or a lack of knuckles (38.5%, 10/26), and syndactyly (34.6%, 9/26). 57.7% (15/26) of the Chinese CdLS individuals had malformations of only the upper extremities.

Furthermore, 92.3% of the 26 Chinese patients diagnosed with CdLS had major malformations, including, in sequence, microcephaly (57.7%, 15/26); cleft palate (57.7%, 15/26); and heart (42.3%, 11/26), genital (38.5%, 10/26), eye (23.1%, 6/26) and dental anomalies (15.4%, 4/26). 88.5% of the subjects (23/26) had cognitive impairment. In addition, 92.3%of the 26 cases (24/26) had the clinical complication of feeding difficulties.

### 3.2 Genetic analysis findings

**Figure 3:**
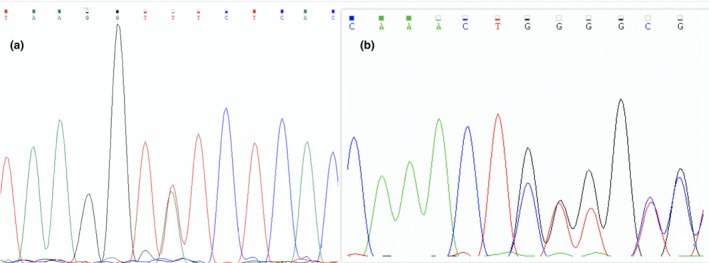
Mutations in *NIPBL* in two CdLS patients. (a) the c.3768 + 3A>T mutation in intron 15 of patient 1. (b) the c.8274_8275 ins CT mutation in exon 47 of patient 2

Whole exome sequencing (Figure 3) showed a heterozygous mutation (c.3768 + 3A>T) in intron 15 of the *NIPBL* gene (NM_133433.3, NG_006987.2 RefSeqGene) in patient 1. In patient 2, a frameshift mutation c.8274_8275 ins CT was detected in exon 47 of the *NIPBL* gene, which has been predicted to cause the replacement of a valine with a tryptophan at residue 2,760 (p. Val2760Trpfs). Furthermore, c.8274_8275 ins CT was suggested to be pathogenic by the Clinvar database. These two mutations have been previously reported (Oliveira et al., 2010; Richards et al., 2015; Teresa‐Rodrigo et al., 2014). None of these subjects had chromosomal abnormalities.

Only 5 previously reported Chinese CdLS patients underwent gene analysis, and 3 cases were found to be carrying heterozygous mutations in the *NIPBL* gene, including c.4321G > T in exon 20, c.6589 + 5G> C in intron 38 and c.7176T > A in exon 42 (Yang, Xu, & Wang, 2017; Zhong, Liang, Liu, Xue, & Wu, 2012).

## 4 DISCUSSION

### 4.1 Clinical characteristics of the Chinese cohort with CdLS

#### 4.1.1 Neonatal features

The male/female ratio in the Chinese cohort of 26 individuals with CdLS was 1:1.6 (10:16). Selicorni et al. (2007) found that this ratio was 1:0.63 (38:24) in their study of 62 Italian patients with CdLS. 50% of the 26 Chinese individuals were diagnosed with CdLS when they were less than one year old. This is consistent with the study conducted by Ramos et al. in which 52% of 101 Italian CdLS patients were diagnosed within the first year of life (Oliver et al., 2010). The proportion of prenatal growth retardation in the group of Chinese patients with CdLS (88.5%) was higher than that in the 62 Italian CdLS patients (56.4%) observed by Selicorni et al. (2007). The specific reason for the disparity between these findings warrants further investigation.

#### 4.1.2 Craniofacial characteristics

Facial abnormalities are pathognomonic for CdLS and constitute the clinical hallmark of the syndrome, which can lead to the initial diagnosis (Boyle, Jespersgaard, Brøndum‐Nielsen, Bisgaard, & Tümer, 2015). Kline et al. (2018) collected auxological parameters of over 500 individuals with CdLS and observed that the most typical facial dysmorphias were long and thick eyelashes (99%), synophrys and hypertrichosis of the brows (98%), thin lips with down‐turned corners (94%), a depressed nasal bridge with anteverted nares (85%), widely spaced teeth and micrognathia (84%), hirsutism (78%) and cutis marmorata (60%). The frequency of the craniofacial abnormalities observed in the study cohort of 26 Chinese individuals with CdLS was consistent with the above findings (Table 2).

**Table 2:** Clinical data of 26 Chinese cases with CdLS

Anomalies and manifestations	No. of cases	Percentage (%)
Craniofacial characteristics		
Microcephaly	11	42.3
Synophrys and hypertrichosis of the brows	24	92.3
Long and thick eyelashes	22	84.6
Depressed nasal bridge with anteverted nares	17	65.4
Long and prominent philtrum	11	42.3
Thin lips with down‐turned corners	19	73.1
Thick and low‐set ears	11	42.3
High palate	15	57.7
Dental anomalies	4	15.4
Micrognathia	9	34.6
Skeletal deformations		
Small hands and feet	13	50.0
Small limbs	2	7.7
The 5th finger clinodactyly	10	38.5
Syndactyly	7	26.9
Hypoplasia of phalanges	11	42.3
Palmar creases	19	73.1
Neurosensory manifestations		
Hearing loss	5	19.2
Ophthalmic abnormalities	2	7.7
Gastrointestinal manifestations	10	38.5
Cardiovascular manifestations	9	34.6
Genitourinary manifestations		
Genital malformations	7	26.9
Urological malformations	1	3.8
Skin manifestations	17	65.4

#### 4.1.3 Limb anomalies

Among the 26 Chinese individuals diagnosed with CdLS, 25 patients of them had some extremity malformations including minor differences in morphologies involving the limbs (e.g., proximally placed thumb, small hand, clinodactyly, syndactyly) (Table 2). This result was consistent with previously described results in the literature, in which 86% of the 310 CdLS individuals observed by Jackson, Kline, Barr, and Koch (1993) and 100% of the 378 CdLS subjects in the large cohort studied by Mehta et al. (2016) exhibited bone anomalies. Furthermore, malformations of the upper extremities in CdLS patients are more common than those of the lower extremities (Mehta et al., 2016). And this is also true for the 26 Chinese CdLS cases: 8 cases had both upper and lower extremities anomalies, 16 cases had only upper extremities anomalies and no cases had only lower extremities manifestations (Table 2).

#### 4.1.4 Intellectual disability

Cognitive impairment with behavioral and neurological problems is a hallmark of CdLS. We found that 23 patients among the 26 Chinese individuals diagnosed with CdLS had impaired psychomotor development and intellectual disabilities, which is consistent with previous studies (Grados, Alvi, & Srivastava, 2017). Srivastava et al. (2014) studied 41 children with CdLS from 5 to 18 years old and found that 17% did not have autism, 41% had mild autism, and another 41% had severe autism based on the Childhood Autism Rating Scale (CARS). Only 2 of the 26 Chinese cases with CdLS were reported to have obvious autism manifestations and this percentage (7.7%) is much lower than the data found by Srivastava et al. This might be attributable to the fact that only 5 cases of the 26 Chinese CdLS patients were older than 5 years old when they were diagnosed as CdLS and the autism features of the rest cases hadn't manifested yet. However, it doesn't mean the behavioral abnormalities in Chinese CdLS cohorts are less common. On the contrary, it might indicate that many elder children with CdLS haven't been diagnosed in China yet which should arouse further attention of clinicians.

#### 4.1.5 Other malformations and manifestations

In addition to craniofacial, skeletal, growth and developmental abnormalities, 26 Chinese CdLS patients also had other malformations and manifestations including hearing loss (19.2%, 5/26), ophthalmic abnormalities (7.7%, 2/26), gastrointestinal (38.5%, 10/26), cardiovascular (34.6%, 9/26), genitourinary (30.8%, 8/26) and skin manifestations (65.4%, 17/26) (Table 2). The other malformations in the Italian cohort studied by Selicorni et al. (2007) included, in sequence, microcephaly (66%, 41/62) and heart (29%, 18/62), genital (25.8%, 16/62), eye (24.2%, 15/62), neurosensory deafness (19.35%, 12/62), dental (17.7%, 11/62), urological (16%, 10/62).

The frequencies of hearing and dental abnormalities among the 26 Chinese CdLS individuals were consistent with those of the Italian cohort. Furthermore, 26 Chinese CdLS cases had higher frequency of cardiovascular manifestations and lower incidence rates of microcephaly, eye and gastrointestinal manifestations than 62 Italian individuals with CdLS studied by Selicorni et al. In addition, among the 26 Chinese CdLS cases, genital anomalies (26.9%, 7/26) had much higher frequency than urological anomalies (3.8%, 1/26). However, in the 62 Italian cohort with CdLS studied by Selicorni et al., the difference between the frequency of genital anomalies (25.8%, 16/62) and urological anomalies (16%, 10/62) was not very obvious. Moreover, the frequency of epilepsy in Italian cohort was 17.7% (11/62), while we didn't find any cases with epilepsy manifestations among the 26 Chinese CdLS individuals. And according to a study of 295 dead CdLS individuals, respiratory diseases including pneumonia as a result of GER were the most frequent cause of death (30.8%, 91/295) (Schrier et al., 2011). It is also confirmed by Oliver et al. (2010) that the most common manifestation in the patients with CdLS was GER, usually resulting in pneumonia, the prevalence of which was approximately 73%. However, only 4 cases in the current study were found to have GER. For the prevalence of GER complicated by CdLS in the Chinese cohort, further studies in larger cohorts are needed.

### 4.2 Genetic analysis

CdLS is a genetically and clinically heterogeneous disorder. DNA sequence variations in *NIPBL, SMC3* and *RAD21* are closely correlated with the autosomal dominant form of CdLS, while *SMC1A* and *HDAC8* abnormalities result in the X‐linked form of CdLS (Kaiser et al., 2014). The mutations in these five genes leading to CdLS are all involved in the cohesin pathway. Cohesin and regulatory cohesin genes are responsible for chromosome segregation, DNA repair, and gene transcription regulation (Liu & Krantz, 2009).

*NIPBL* gene mutations have been found to be the most common causes of CdLS worldwide and contribute to approximately 50% of all CdLS cases, while *SMC1A* and *SMC3* alterations were estimated to account for 5% and < 1%, respectively (Oliveira et al., 2010). Reports of the occurrence of mutations in the *RAD21* and *HDAC8* genes are rather anecdotal; thus, further studies in larger cohorts are needed for assessment (Kline, Barr, & Jackson, 1993).

**Figure 4:**
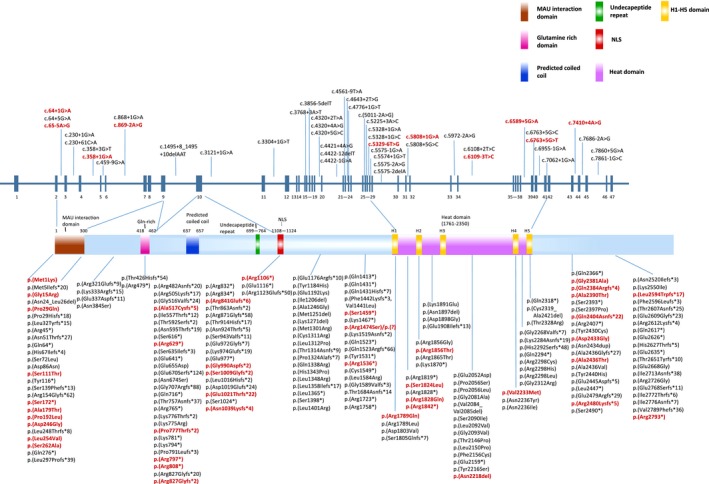
Schematic representation of mutations identified in *NIPBL* gene within *NIPBL*‐LOVD database (last accessed July 2017). The 47 exons of *NIPBL* are indicated with blue bars. Positions of all mutations are drawn to scale along the protein product of the longest isoform. The domains are marked by blocks with different colors. Mutations reported several times in the database are represented with red in bold

The *NIPBL* gene is located at 5p13.2; this gene is composed of 47 exons and encodes two isoforms of delangin (A and B, with 2,804 and 2,697 amino acids, respectively). Delangin plays a key role in ensuring the appropriate development of organs in the growing embryo. *NIPBL* consists of different domains, including an N‐terminal MAU interaction domain (1–300), a glutamine‐rich domain (418–462), an undecapeptide repeat (699–764), a predicted nuclear‐localization signal (NLS, 1108–1124), and a conserved domain with five HEAT repeats (H1: 1,767–1,805, H2: 1,843–1,881, H3: 1,945–1,984, H4: 2,227–2,267, H5: 2,313–2,351) (Mannini et al., 2013). To date, the *NIPBL*‐LOVD database (Leiden open variation database) describes 266 unique DNA variants, reported in 352 individuals (last accessed July 2017). These mutations include substitutions (65.1%, 229/352), deletions (23.9%, 84/352), duplications (8.8%, 31/352), insertions/deletions (1.4%, 5/352) and insertions (0.9%, 3/352) (Figure 4). However, as only 7 cases in the current analysis underwent genetic screening, further studies in larger cohorts are needed to determine the type and frequency of *NIPBL, SMC3, RAD21, SMC1A* and *HDAC8* gene mutations in Chinese patients with CdLS.

## 5 CONCLUSIONS

Herein, we present 2 unrelated Chinese CdLS patients. Furthermore, we are the first to apply the consensus criteria of clinical phenotype in CdLS patients proposed by Kline et al. in 2018 to Chinese patients with CdLS. We observed that the clinical data and gene variants in all 26 Chinese cases with CdLS collected by literature review did not significantly vary from those of other populations. This is the first time that a summarization of clinical and genetic characteristics of Chinese patients with CdLS has been reported. However, further studies in larger cohorts are needed to elucidate the genotype‐phenotype correlations and the prevalence of variable *NIPBL* mutations in Chinese populations with CdLS.

## CONFLICT OF INTEREST

There is no conflict of interest.

## AUTHOR CONTRIBUTION

Huijuan Zhu and Hui Pan contributed equally to the current research paper. These two authors are both corresponding authors. Shuo Li was responsible for the formal analysis, writing and editing the manuscript. Hui Miao contributed to collecting clinical and genetic materials. Hongbo Yang and Linjie Wang were responsible for funding acquisition. Fengying Gong and Shi Chen contributed to methodology and supervision. This article has not been published elsewhere in whole or in part. All authors have approved the content and agreed to submit for consideration for publication in the journal.

## DECLARATION OF PATIENT CONSENT

The authors certify that they have obtained all appropriate patient consent forms. In the form, the patients have given their consent for their images and other clinical information to be reported in the journal. The patients understand that their names and initials will not be published and due efforts will be made to conceal their identity.

## COMPLIANCE WITH ETHICAL STANDARDS

Examination protocols were approved by the ethics committees of Chinese Academy of Medical Science and Peking Union Medical College with the following reference number: JS‐1663.
