# A report of 2 cases of Cornelia de Lange syndrome (CdLS) and an analysis of clinical and genetic characteristics in a Chinese CdLS cohort

**DOI:** 10.1002/mgg3.1225

**Published:** 2020-05-03

**Authors:** Shuo Li, Hui Miao, Hongbo Yang, Linjie Wang, Fengying Gong, Shi Chen, Huijuan Zhu, Hui Pan

In Li et al. (2020), the patients’ eyes in Figure 1 should have been hidden when published. Below is the updated figure:

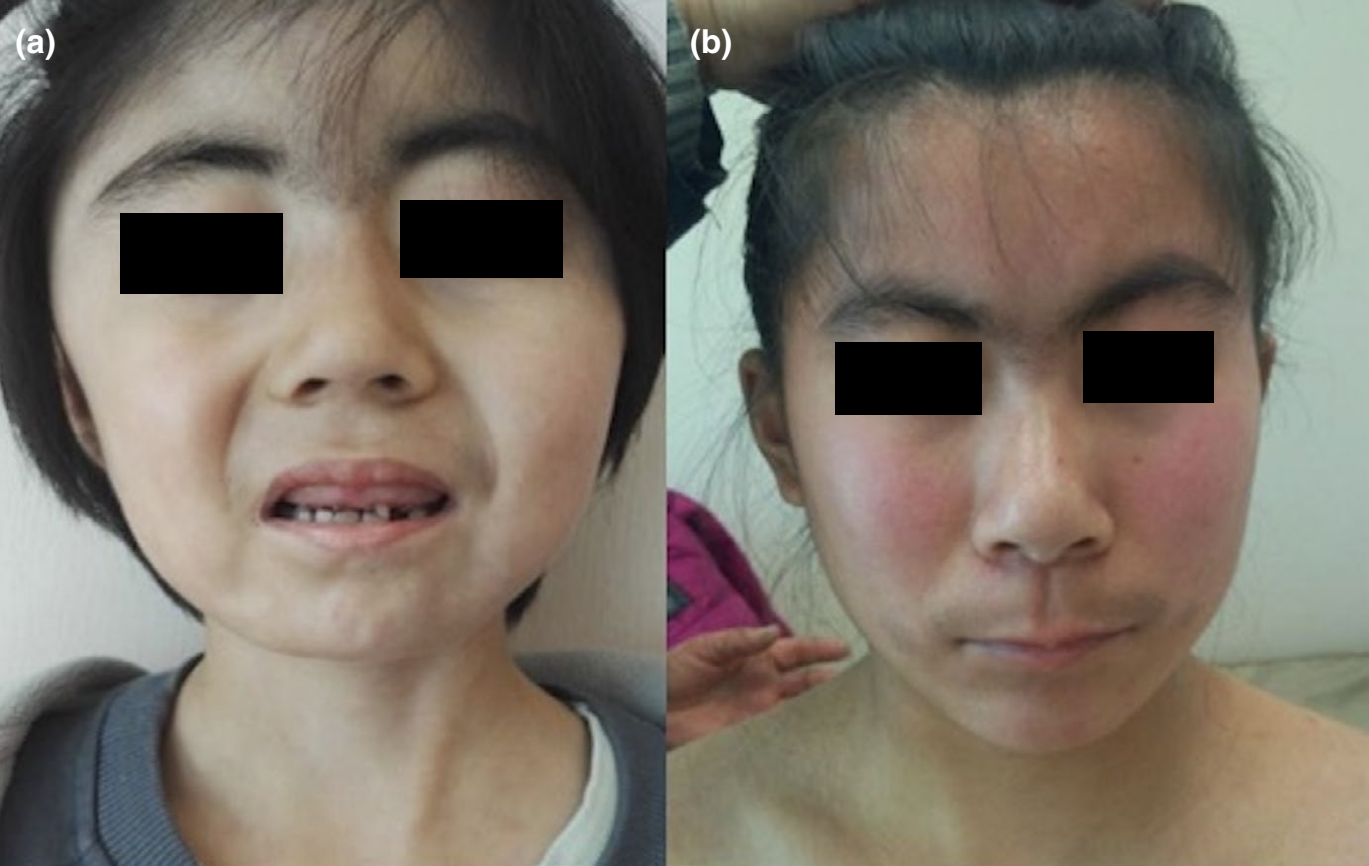

This has been corrected online.
